# ABO blood group antigens in saliva - A cross sectional study

**DOI:** 10.6026/973206300210329

**Published:** 2025-03-31

**Authors:** Surekha S., Saraswathi Gopa K., Harshavardhan B.G, Swathi T.

**Affiliations:** 1 Department of Oral medicine and radiology, Meenakshi Ammal Dental College and Hospital, Chennai, Tamilnadu, India

**Keywords:** ABO blood groups, secretors, saliva, ABH blood group antigens, agglutination

## Abstract

Blood group antigens are present on red blood cells and in bodily fluids like saliva, making them useful in forensic investigations when blood or tissue samples are unavailable. Therefore, it is of interest to determine the secretory status of A, B, H antigens in saliva to correlate it with blood samples. A prospective, randomized, cross-sectional study of 102 individuals was conducted, with saliva collected using drooling and gauze methods. Blood group was determined using slide agglutination. A strong correlation (Pearson coefficient r = .980**) was observed between ABH antigens in saliva and blood group as well as with dry salivary sample (p = 0.04*). Thus, the feasibility and reliability of identifying ABO blood group antigens in saliva, supporting its potential as a non-invasive alternative for forensic and clinical applications is shown.

## Background:

Blood is considered as one of the important evidence in crime scenes and disasters because once the blood group is determined it is unchanged throughout the life [1, 2] approximately 78% of all individuals possess the secret or gene that governs the secretion of water soluble ABH antigens into all body fluids except cerebrospinal fluid such secreted antigens can be demonstrated in saliva [3]. A person is considered as a secret or if they release their blood type antigens into bodily fluids such as saliva and mucus. On the other hand, a non-secretor does not release, or releases very little, of their blood type antigens into bodily fluids [4, 5]. Therefore, it is of interest to evaluate the ABH secretor status from saliva and identify the most effective method for detecting blood group antigens in secretors' saliva [6].

## Materials and Methods:

In Two methods Saliva was collected from subjects for resting saliva it is collected using the drooling method, gathering 5 to 10 ml in a sterile container. It was centrifuged at 900-1000 rpm for 8-10 minutes, then the supernatant was transferred to a clean test tube and boiled(Water bath) for 8-10 minutes to inactivate enzymes. The saliva was re-centrifuged at 900-1000 rpm for 8-10 minutes to obtain a clear supernatant, discarding any opaque or semisolid material. And for dry salivary sample, Saliva was collected by placing gauze in the mouth, then placing the gauze in a sterile container to dry for an hour. Afterward, 4ml of distilled water was added and left overnight. The dried salivary sample was then transferred from the container to an Eppendorf tube.

## For blood group determination:

Capillary blood was obtained from the patient by pricking the ring finger and using the slide agglutination method.

## Materials required:

## Reagents:

[1] Anti A and Anti B

[2] Anti H

[3] A and B red cells and Group O cells

[4] Frozen or fresh saliva from persons known to be secretors or non-secretors.

## For blood group antigens in saliva:

## In resting saliva:

## Zero dilution method:

0.50 ml of saliva sample was taken with 0.50 ml of A B H reagent in different Eppendorf tubes. After incubating it for 10 minutes, 0.20 ml of pooled blood cells (A B O) were added to the appropriate Eppendorf tubes. Then, they were incubated again for 1 hour and agglutination was checked by visual inspection.

## With dilution method:

0.50 ml of saliva was added with 1:10 diluted reagent after incubating for 20 minutes. Subsequently, 0.20 ml of pooled blood cells (A, B, O) was added to appropriate Eppendorf tubes, After that, the mixture was incubated for an hour. Finally, agglutination was checked by visual inspection.

## In dry salivary sample:

The dry salivary sample was placed in a hot water bath for 10 minutes. Next, 0.50 ml of the dry sample saliva was transferred into a separate Eppendorf tube and then the 1:10 reagent was added. After that, the mixture was incubated for ten minutes. Following this, 0.20 ml of pooled blood cells was added to the appropriate Eppendorf tubes. After another 10 minutes of incubation, agglutination was checked by visual inspection.

## Interpretation:

[1] If indicator red cells agglutinate in tubes containing saliva, it indicates that the saliva does not contain the corresponding antigen.

[2] If known antibodies fail to agglutinate indicator red cells after incubation with saliva, it indicates that the saliva contains the corresponding antigen.

## To determine Rh factor:

Anti D reagent was added to the saliva sample and it was incubated for 20 minutes. Subsequently, 0.20 ml of pooled blood cell (O) was added and the mixture was incubated for 1 hour. Agglutination was checked by visual inspection.

## Interpretation:

[1] Agglutination Seen in Negative Rh

[2] Agglutination Not seen in Positive Rh

## Results and Discussion:

Determining blood groups is essential for identifying individuals and ruling out potential identities or lineages, especially in legal and forensic contexts 7]. The importance of blood group determination lies in the unchanging nature of blood groups remaining constant over a person's lifetime [8, 9 and 10]. Indirect blood grouping such as through saliva analysis has become crucial in forensic investigations, aiding in identifying suspects in criminal cases [11]. Besides its forensic applications, this method has wider implications across different fields [12]. Its non-invasive nature makes it particularly promising for use in pediatric and geriatric populations [13] and where conventional blood collection methods may pose challenges. In our study, two different methods were used to detect ABO blood group antigens in resting saliva, include the With Dilution Method, Zero Dilution Method with Samples and In Dry salivary samples. Among these three methods, With Dilution Method proved to be more effective in identifying blood group antigens compared to the Zero Dilution Method. Additionally, the dry salivary sample method was found to be more convenient in sample collection compared to the other methods.

Nevertheless, it is noteworthy that the incubation period for this method was significantly longer than that of the other methods. The study examined 102 individuals and found the following blood group distribution (Figure 6 - see PDF), 20 individuals had blood group A as shown in (Figure 1 - see PDF), 32 individuals had blood group B has shown in (Figure 2 - see PDF), 40 individuals had blood group O has shown in (Figure 3 - see PDF) and 10 individuals had blood group AB as shown in (Figure 4 - see PDF). Regarding the Rh factor, 100 individuals were Rh-positive, whereas only 2 individuals were Rh-negative. Additionally, the study investigated blood group determination in saliva samples, uncovering those 99 individuals were secretors, while 3 individuals were non-secretors (Figure 7 - see PDF) and the results of the study are (Table 1) descriptively showing the total number of samples in each blood group, AB+ve (10 samples), A+ve (20 samples), B+ve (29 samples), O+ve (41 samples), O-ve (1 sample), B-ve (1 sample) respectively. The samples were collected from both male and female participants. (Table 2) descriptively showing the secretory status in salivary sample according to each blood group.

The detection of secretor status is displayed in frequency (*i.e.*, percentage value). 90% of individuals in AB+ve blood group, 90% in A+ve blood group, 100% in B+ve, B-ve, O+ve and O-ve Blood group were detected.

(Table 3)(Figure 5 - see PDF) *Pearson correlation coefficient. 980** shows strong correlation between ABH antigen in salivary sample to the blood group of an individuals. P-value <0.001*** shows very high statistical significance. (Table 4) shows the intergroup comparison of blood group determined in terms of saliva, obtained from three different modalities. Dry group sample estimation found to be statistically significant on comparison with group 2 with p-value of 0.04*. On similar comparison between Dry group and group 3, Group 2 and group 3, it is found to show no statistically significant difference. These findings offer valuable insights into the distribution of blood groups and secretor status among the study population, enhancing our understanding of genetic and physiological variations among individuals. The 95% secretor rate found in this study is higher than the results found in studies by Tejasvi *et al.* (2021) [14], Badiye *et al.* (2013) [15], Thrumiaya *et al.* (2017) [16] and Motghare *et al.* (2016) [17].

According to a 2017 study by Thrumiaya *et al.* people with blood types A and O, regardless of gender, had a 100% secretor status. The importance of determining one's ABO blood group from saliva as a useful tool in quantitative analysis is highlighted by the identification of blood group antigens in a variety of physiological fluids, including saliva. Our result is accordance with the study's conclusion that the absorption-inhibition method is superior for detecting secretor status. Velani *et al.* [18] 2018 study revealed a 100% positive correlation for ABO blood grouping but only a 14.81% positive correlation for Rh typing between the blood from the extraction socket and dried salivary samples. This is accordance with our study.

Tejasvi *et al.* (2021) [14], 52 out of 60 subjects had antigen secretors in their saliva, with a percentage of 86.66% and 8 subjects did not have antigen secretors (13.33%). slightly greater percentages of secretor status (84.6%) in men and 88.2% in women. Evaluation of blood type antigen secretor status from saliva using the absorption inhibition approach can be valuable technique accordance with our study. Rajawat *et al.* (2023) in 282 subjects out of this Rhesus positive were 94% Rhesus negative were 6% which is accordance with our study [19]. Metgud *et al.* (2016) In their study blood group A and O revealed 100% secretor status for both males and females, B and AB group revealed 95% secretor status which is not accordance with our study in our study AB & A group were 90% secretor others were 100 % secretor status among 102 individuals [20]. In the Sen *et al.* (2015) study, out of the 100 samples analyzed, 77 were found to be secretors and 23 to be non-secretors. Out of the 77 samples, 62 produced results that were consistent with blood grouping when the absorption inhibition approach was applied. In contrast, 70 out of 77 samples that used absorption elution produced findings that matched. Notably, the absorption elution method showed improved sensitivity, which is not accordance with our study [21].

## Conclusion:

The feasibility and reliability of identifying ABO blood group antigens in saliva, indicating the possibility of using saliva-based testing as a non-invasive substitute for traditional blood-based techniques is shown. The identification of ABO blood group antigens in saliva has great potential for use in forensic applications. It can be a valuable tool for identifying individuals in forensic investigations, especially in legal contexts or crime scenes as it is easy to use and has a non-invasive collection process.

## Financial support and sponsorship:

Nil

## Figures and Tables

**Table 1:** Descriptive tables showing the blood group in sample obtained from participants.

**Blood groups**	**Total number of samples**	**Males**	**Female**
AB+ve	10	3	7
A+ve	20	6	14
B+ve	29	11	18
O+ve	41	11	30
O-ve	1	0	1
B-ve	1	1	0

**Table 2:** Secretory status in salivary sample according to each blood group and their percentage

	**secretor**	**non secretor**	**percentage**
AB+ve	9	1	90
AB-ve	0	0	0
A+ve	18	2	90
A-ve	0	0	0
B+ve	29	0	100
B-ve	1	0	100
O+ve	41	0	100
O-ve	1	0	100

**Table 3:** Correlation assessment of ABH antigen in salivary sample to blood groups

**slide agglutination method**		
blood group determination in saliva	Correlation	.980**
	Sig. (2-tailed)	0
	N	102
	**. Correlation is significant at the 0.01 level (2-tailed).	
*Pearson correlation coefficient .980**
shows strong correlation between
ABH antigen in salivary sample to the blood group of an individuals.
P-value <0.001*** shows very high statistical significance

**Table 4:** Intergroup comparison of blood group determined in terms of saliva, obtained from three different modalities

**Dependent Variable**			**Mean Difference (I-J)**	**Std. Error**	**Sig.**	**95% Confidence Interval**
						**Lower Bound**	**Upper Bound**
Blood group determined in saliva	Dry group	group 2	-.64706*	0.25668	0.04	-1.2722	-0.022
	group 3	-0.32353	0.25668	0.63	-0.9486	0.3016
	group 2	group 1	.64706*	0.25668	0.04	0.022	1.2722
		group 3	0.32353	0.25668	0.63	-0.3016	0.9486
	group 3	group 1	0.32353	0.25668	0.63	-0.3016	0.9486
	group 2	-0.32353	0.25668	0.63	-0.9486	0.3016
*. The mean difference is significant at the 0.05 level.
P-value <0.01* shows high statistical significance
Post hoc analysis - Bonferroni pairwise comparison
